# Effectiveness and neural mechanisms of home-based telerehabilitation in patients with stroke based on fMRI and DTI: A study protocol for a randomized controlled trial

**DOI:** 10.1097/MD.0000000000009605

**Published:** 2018-01-19

**Authors:** Jing Chen, Mingli Liu, Dalong Sun, Yan Jin, Tianrao Wang, Chuancheng Ren

**Affiliations:** a Department of Neurology, Shanghai Fifth People's Hospital, Fudan University, 801 Heqing Road, Minhang District; b Division of Gastroenterology, Department of Internal Medicine, Zhongshan Hospital Affiliated to Fudan University, Xuhui District; c Department of Rehabilitation; d Department of Radiology, Shanghai Fifth People's Hospital, Fudan University, Minhang District; e Departments of Neurology, Shanghai East Hospital Affiliated to Tongji University, Pudong New Area, Shanghai, China.

**Keywords:** DTI, fMRI, motor cortex, motor recovery, stroke, telerehabilitation

## Abstract

**Background:**

Stroke is one of leading diseases causing adult death and disability worldwide. Home-based telerehabilitation has become a novel approach for stroke patients as effective as conventional rehabilitation, and more convenient and economical than conventional rehabilitation. However, there is no study assessing the mechanism of home-based telerehabilitation in promoting motor recovery among stroke patients with hemiplegic.

**Aims:**

This study is designed to determine the efficacy and explore the mechanism of motor recovery after home-based telerehabilitation in stroke patients with motor deficits.

**Methods/Design:**

In a single-blinded randomized controlled pilot study, patients with acute subcortical stroke (n = 40) are assigned to receive home-based telerehabilitation or conventional rehabilitation. Task-based or resting-state functional magnetic resonance imaging (rs-fMRI), diffusion tensor imaging (DTI), and Fugl–Meyer assessment (FMA) score will acquired before and after rehabilitation. Activation volume of bilateral primary motor (M1), supplementary motor area (SMA), premotor cortex (PMC); lateralization index (LI) of interhemispheric M1, SMA, and PMC; functional connectivity of bilateral M1, SMA, PMC; fractional anisotropy (FA) will be measured; correlation analyses will be performed between neuroimaging biomarkers and FMA score pre- and postrehabilitation.

**Discussion:**

We present a study design and rationale to explore the effectiveness and neural mechanism of home-based rehabilitation for stroke patients with motor deficits. The study limitations related to the small-amount sample. Moreover, home-based rehabilitation may provide an alternative means of recovery for stroke patients. Ultimately, results of this trial will help to understand the neural mechanism of home-based telerehabilitation among stroke patients with hand movement disorder.

## 1 Background

Stroke is characterized by its high morbidity, disability, and mortality rate, which brings serious damage on human health.^[1,2]^ Statistics data revealed that the disability rate of stroke can reach up to 60% to 80%, enormously decreasing quality of life for survivals.^[3,4]^ Numerous studies showed that patients who undertook early rehabilitation treatment can to some extent have a lower disability rate and better improvement of daily living activities.^[5–7]^ However, majority of stroke patients have no access to effective rehabilitation due to the limits of traditional rehabilitation sources under the current state of health care construction in China. Furthermore, the therapeutic effects of rehabilitation treatment in stroke patients are poor because of relative high costs of traditional rehabilitation and the difficulty in adhering to rehabilitation treatment on account of the fatigue of commuting rehabilitation training institution and family.^[8]^ Telerehabilitation could facilitate the access to rehabilitation treatment, no matter where and when, improving patients’ compliance and chances of recovery.^[9,10]^

Task-based functional magnetic resonance imaging provides an effective approach to studying brain active region.^[11]^ Longitudinal task-based fMRI measurements could provide qualitative, quantitative, and dynamic information about conditions of remaining neurons during the process of recovery, including monitoring activation state of each related cerebral cortex qualitatively and dynamically, as well as measuring activation volume of motor area quantitatively and dynamically.^[12]^ Resting-state functional magnetic resonance imaging (rs-fMRI) is widely applied to investigate integration network of brain function.^[13]^ It could dynamically reflect the changes of brain motor function network connection.^[14]^ Diffusion tensor imaging (DTI) is a noninvasive method used to study microstructure, providing dynamic information about the recovery state of lesioned brain tissue qualitatively and quantitatively.^[15]^

Our previous research and other numbers of studies have shown that acute stroke patients undertaking home-based rehabilitation could achieve equal effects compared with traditional rehabilitation, in terms of effectiveness of neurologic function improvement, degree of stress relief of caregivers and safety of rehabilitation implementation.^[16–18]^ To further explore the mechanisms of home-based telerehabilitation in improving neurological functions of stroke patients, our study investigated dynamic changes of motor activated region, changes of motor functional connectivity, and structural changes of white matter fiber in patients with motor deficits by means of task-based fMRI, rs-fMRI, and DTI.

We designed this study to provide more robost evidence of the application of home-based rehabilitation on patients of stroke with motor deficits.

### 1.1 Methods/design

#### 1.1.1 Study design

**Figure 1:**
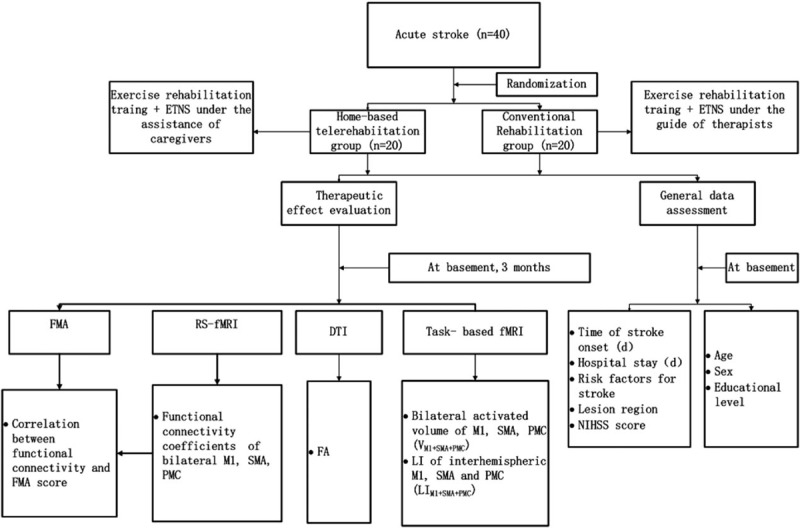
The flow scheme of study.

The trial is an assessor blinded, paralleled randomized controlled trial (RCT). The overall flowchart is shown in Figure 1. The main aim is to explore the mechanisms of home-based telerehabilitation on improving neurological functions of stroke patients. The secondary objective is to determine the effects of home-based telerehabilitation in stroke patients.

### 1.2 Ethics approval and consent to participate

This trial follows the principal ethical guidelines for conducting clinical trials of Helsinki Declaration and has received approval from the Independence Ethics Committee of the Fifth Peoples’ Hospital of Shanghai, Fudan University (ethic approval number: 2014-ETRE-066) and has been registered in the Chinese Clinical Trial Registry (ChiCTR-IPR-17011757). Any issues related to clinical study safety will be reported to the Ethics Committee in time, such as the changes of clinical protocols or patient information. Participants will be acquainted with informed consent and asked to sign for it before being recruited.

### 1.3 Randomization and blinding

#### 1.3.1 Randomization

In this pilot study, we aim to collect 40 patients diagnosed as stroke from the Department of Neurology, Shanghai the Fifth People's Hospital, Fudan University. Patients included in the study will be randomly allocated into 2 groups in a ratio of 1:1 after baseline assessment: home-based rehabilitation group and conventional rehabilitation group. An independent statistician will conduct randomized allocation by using a computer. The former will receive exercise training rehabilitation and electromyography-triggered neuromuscular stimulation with the assistance of caregivers at home. Specialist physicians will evaluate the recovery condition of patients via telerehabilitation system every week and give guidance on rehabilitation training and electro-stimulation therapy. The latter will receive the same rehabilitation training under the guide of specialist physicians face to face. Specialist physicians will evaluate the recovery condition of patients at outpatient clinic every week and adjust the rehabilitation strategies. Allocation concealment will be guaranteed, since allocation information will be protected in opaque sealed envelopes by a specially assigned person who is not involved in the study.

#### 1.3.2 Blinding

In this study, a specific device during home-based telerehabilitation will be adopted; the patients and home-based rehabilitation therapists cannot be blinded. To ensure the research quality, the assessors and data analysts are totally blind to allocation and rehabilitation intervention until they finish all the data collection and analyses.

### 1.4 Participants

#### 1.4.1 Inclusion criteria

1. First-ever stroke and in stable condition after acute management of ischemic stroke.
2. Aged between 35 and 85, male or female.
3. Responsible focuses of the unilateral white matter area indicated by cranial CT or MRI.
4. No more than 14 days from stroke onset, with unilateral limb movement dysfunction and be proved to be right handed after standardized testing.
5. National Institute of Health Stroke Scale (NIHSS) scores from 2 to 20, Grading Muscle Strength of paralyzed upper limbs between grade 1 and grade 4, and have not received any standard rehabilitation intervention before inclusion.
6. Sign the informed consent by the patient himself or voluntarily.

#### 1.4.2 Exclusion criteria

1. Glasgow Coma Scale (GSC) scores less than 15, have dementia indicated by Minimum Mental State Examination (MMSE) assessment, have mental disorders and be unable to cooperate with examination or treatment.
2. Motorial and sensory disturbance not induced by stroke or induced by previous ischemic stroke.
3. Serious comorbidity like malignant tumor, primary cardiac, hepatic, renal or hematopoietic system diseases.
4. History of cognitive disorder, psychiatric disorders, drug abuse and alcoholism.
5. Infections or ruptures on the skin of forearms or legs.
6. Have a heart pacemaker, metal stent, plate or an implant that is susceptible to the current pulse in the body.
7. Be pregnant or lactant or have birth plans recently.
8. Be long-term musical conductors or keyboard operators before stroke onset.
9. Have claustrophobia.
10. Be unable to finish basic course, or be hard to maintain compliance and follow-up.

### 1.5 Intervention

Intervention measures were described in detail in our previous studies.^[19]^ Rehabilitation therapies include exercise rehabilitation training and electromyography-triggered neuromuscular stimulation (ETNS).

#### 1.5.1 Exercise rehabilitation training

All patients will receive Brunnstrom motor function grading evaluation by the same rehabilitation therapist after discharged from hospital. Individualized rehabilitation training plans will be made based on specific conditions of each patient, including physical therapy (PT) and occupational therapy (OT). Patients will be given corresponding treatment based on the Brunnstrom stages. Patients receive the designated exercise rehabilitation for 45 minutes, once a day for 5 days a week for 3 months.

#### 1.5.2 ETNS

ETNS is a novel method developed from the combination of myoelectric biofeedback technology and neuromuscular electrical stimulation (NMES), which has outstanding features compared to NMES. It can increase stroke patients’ initiatives in taking rehabilitation training and effectively improve motor function of the affected limb and ability of daily living, so it has becomes a new therapeutic option in treating paralyzed patients. Patients in both groups will receive ETNS 20 minutes each time, twice a day for 5 days a week for 3 months. The MyoNet-AOW biofeedback equipment used in our study has been approved by China Food and Drug Administration (Shanghai food drug supervision character 2010 no. 221114).

### 1.6 Outcomes

#### 1.6.1 Primary outcome

Primary outcomes will be conducted before and after rehabilitation therapies, including sum of bilateral activated volume of primary motor (M1), supplementary motor area (SMA), premotor cortex (PMC) (V_M1+SMA+PMC_); lateralization index (LI) of interhemispheric M1, SMA, and PMC (LI_M1+SMA+PMC_)(LI = (ΣV_C_ − ΣV_I_)/(ΣV_C_+ ΣV_I_), subscripts C and I refer to contralateral and ipsilateral hemisphere responses, respectively. The ΣV measures the regional activation level associated with the activated voxel); functional connectivity of bilateral M1, SMA, PMC, and the value of fractional anisotropy (FA) in corticospinal tract (CST) at ipsilesional posterior limb of internal capsule. Data will be collected before (at basement) and after (3 months) rehabilitation.

#### 1.6.2 Secondary outcome

Fugl–Meyer assessment (FMA) score will be assessed as secondary outcome. The FMA score consists of 17 items, ranging from 0 to 34, with lower scores demonstrating poorer movement function.

### 1.7 Data acquisition and preprocessing

Data will be acquired by a Siemens 3.0T Magnetom Trio scanner (Siemens Medical Solutions). Subjects will receive 2 MRI sessions (1 before and 1 after rehabilitation therapy). Each session included 1 rs-fMRI scan, 1 task functional scan and 1 DTI scan. For fMRI scan, parameters are TR/TE/FA = 1500 ms/30 ms/90°, 200 time points, resolution = 3.4 × 3.4 × 5 mm^3^, 21 axial slices. During rs-fMRI scan, patients will be asked to keep away with the eyes closed and think of nothing. The motor task scans used a block design paradigm consisting of alternating 30-seconds periods of performance of an isometric precision grip task and rest for 4 minutes. DTI images will be acquired with the following parameters: matrix size = 94 mm × 94 mm, field of view = 240 mm × 240 mm, 2.6 mm slice thickness, 56 slices. We acquired 1 set of 30 diffusion weighted images with a *b*-value = 1000 s/mm^2^, and 5 no diffusion weighted images.

All data will be analyzed using Functional MRI of the Brain (FMRIB's) Software Library (FSL) tools. Functional data sets will undergo slice timing correction, motion correction, scaling to percent signal change, smoothed with a Gaussian kernel of 8 mm full-width-at-half maximum, bandpass temporal filtering (0.01–0.08 Hz), and grand mean intensity normalized. A whole brain analysis of white matter integrity between groups will be performed to investigating FA. FA quantifies the degree to which water diffusion will be restricted and calculated based on the diffusion tensor. The data will be corrected for head motion and eddy current distortion, and brain extracted using Brian Extraction Tool. FA images will be then created by fitting the data to a tensor model at each voxel using DTI fit within the FMRIB's Diffusion Toolbox. Lesion will be masked out in each subject's data so that all analyses are performed on nonlesioned data.

### 1.8 Definition of ROI and lesion

We choose three pairs of seeds in sensorimotor network, the bilateral primary motor (M1), supplementary motor area (SMA), and premotor cortex (PMC). Hand movement is most closely related to M1.^[20,21]^ The early involvement of the SMA in the process of stroke recovery and correlation of initial activation of the SMA with motor recovery were demonstrated.^[22,23]^ PMC might influence other cortical areas in the surviving motor network to support residual motor output and higher order processes required for motor function.^[24]^ A 6-mm radius sphere will be placed at the above 3 pairs of seeds to generate the regions of interest (ROI).

Lesions will be drawn in each patients’ structural space on the T1-weighted images using the co-registered T2-weighted diffusion images as a reference for lesion extension. The lesion mask is nonlinearly registered into standard space using FMRIB's Non-linear Registration Tool (FNIRT) with an affine transformation (12 degrees of freedom). The lesion mask will be also used during the registration steps of the structural, resting state fMRI, and DTI data so that voxels in the lesioned area would be excluded from the normalization procedure.

### 1.9 Data analyses

Data analysis will be handled by independent statisticians who are blind to the groups. The statistical tests will be 2-side and at the 5% significance level. Means and standard deviation will be used for statistical description of continuous variables. For fMRI data, independent sample *t*-test or independent sample rank test will be used for grouped comparison of quantitative data. Pearson correlation coefficients (*r*) will be used to determine whether patients’ functional connectivity correlated with their FMA score and be computed after application of Fisher's *r*-to-*z* transform to yield variates that are approximately normally distributed (*z* = 0.5 ln[(1+*r*)/(1−*r*)]). All the statistical analysis will be carried out by the SPSS software (Armonk, NY). For DTI data, statistical analyses will be performed using nonparametric permutation testing (Randomize in FSL) with 5000 Monte Carlo simulations. We looked for differences in FA between the groups accounting for age, time of stroke onset, and NIHSS as regressors of no interest in the general linear model.

### 1.10 Quality control

The researchers will receive specific training about protocols before the implementation of the study. They will try their best to make the subjects have a thorough understanding of the trial requirements, so that the subjects could cooperate better during the trial. The related rehabilitation equipment and examinations should be free to the subjects. The medication compliance of the subjects will be supervised by researchers. Superiors appointed by the sponsor will inspect and visit the process of the study at regular intervals to check the original data to ensure its accordance with the content on the case report form (CRF).

### 1.11 Adverse events

Adverse events are unavoidable in the course of rehabilitation treatment. Conditions may appear in few patients during the treatment period, such as unintentional injury, rupture and bleeding of skin, muscle soreness, joint pain, and skin rash. Any adverse events (AE) during the course of rehabilitation treatment should be recorded about time of occurrence, severity, relationship of duration with intervention, and prognosis, which should be recorded in the specific CRF. Researchers should take necessary measures to deal with all AE to ensure the safety of subjects and follow-up the subjects until their physical conditions come back to normal level.

### 1.12 Dissemination policy

Results of the research will be published in peer-reviewed journals and the publications will be on “open access” terms, with datasets will be accessible to the research investigators and statistical assessors. The research results will also be disseminated to the participants, stakeholders, and policymakers (Shanghai Municipal Commission of Economy and Informatization, Shanghai Municipal Commission of Health and Family Planning). The investigators will be responsible for the publication of the research to share results with broader scientific community, regardless of the magnitude or direction of effect.

## 2 Discussion

We present a study design and rationale to explore the effectiveness and neural mechanism of home-based rehabilitation for stroke patients with motor deficits based on fMRI and DTI imaging. A total of 40 stroke patients will be eligibly enrolled in this clinical trial. The primary outcomes involve neural biomarkers; the secondary outcome is FMA to assess motor function; correlation between functional connectivity and motor function will also be calculated. Home-based telerehabilitation may provide an alternative means of recovery for stroke patients. Results of this trial will help to understand the neural mechanism of home-based telerehabilitation among stroke patients with hand movement disorders.

## 3 Author contributions

CCR conceptualized the study, and drafted and revised the manuscript. JC, MLL, and DLS drafted and revised the manuscript, and YJ contributed to the rehabilitation therapies, TRW contributed to the neuroimage methods and statistical analysis. All authors revised the manuscript and contributed to the writing.
